# Supplementary material for: DMRforPairs: identifying Differentially Methylated Regions between unique samples using array based methylation profiles
Source: BMC Bioinformatics. 2014 May 15;15:141. doi: 10.1186/1471-2105-15-141 (PMC4046028; doi:10.1186/1471-2105-15-141)
Supplement: Additional file 2 — DMRforPairs output for the comparison of A431-MCF7 and NA17018-NA17105. Please start from the HTML files in each folder. Available via the BMC Bioinformatics website. [file 1471-2105-15-141-S2.zip › 1394847754114233_MOESM2_ESM/A431_MCF7/figures/1380.pdf]

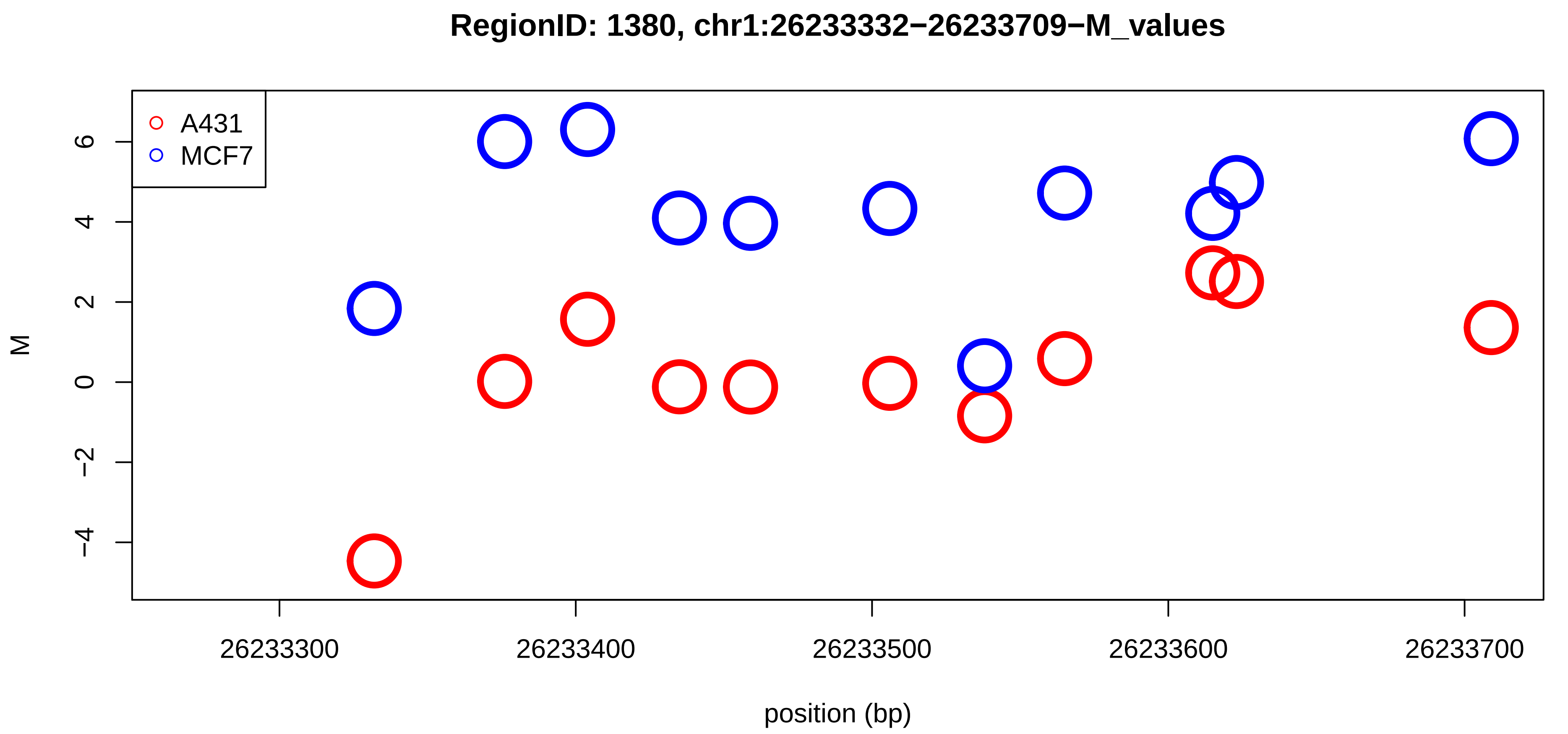

RegionID: 1380, chr1:26233332−26233709−M_values
A431
MCF7
M
6
4
2
0
−2
−4
26233300
26233400
26233500
26233600
26233700
position (bp)

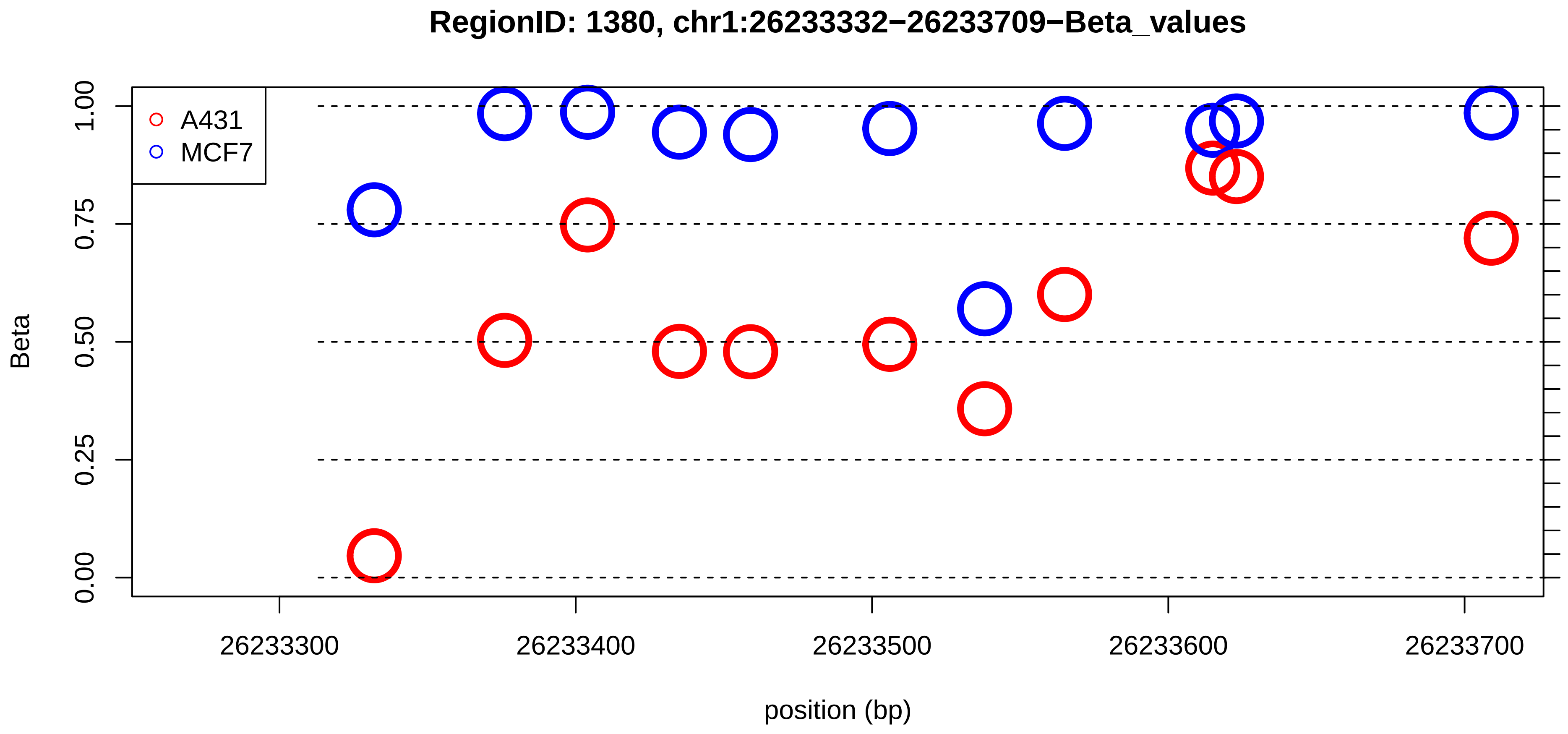

RegionID: 1380, chr1:26233332−26233709−Beta_values
A431
MCF7
Beta
1.00
0.75
0.50
0.25
0.00
26233300
26233400
26233500
26233600
26233700
position (bp)

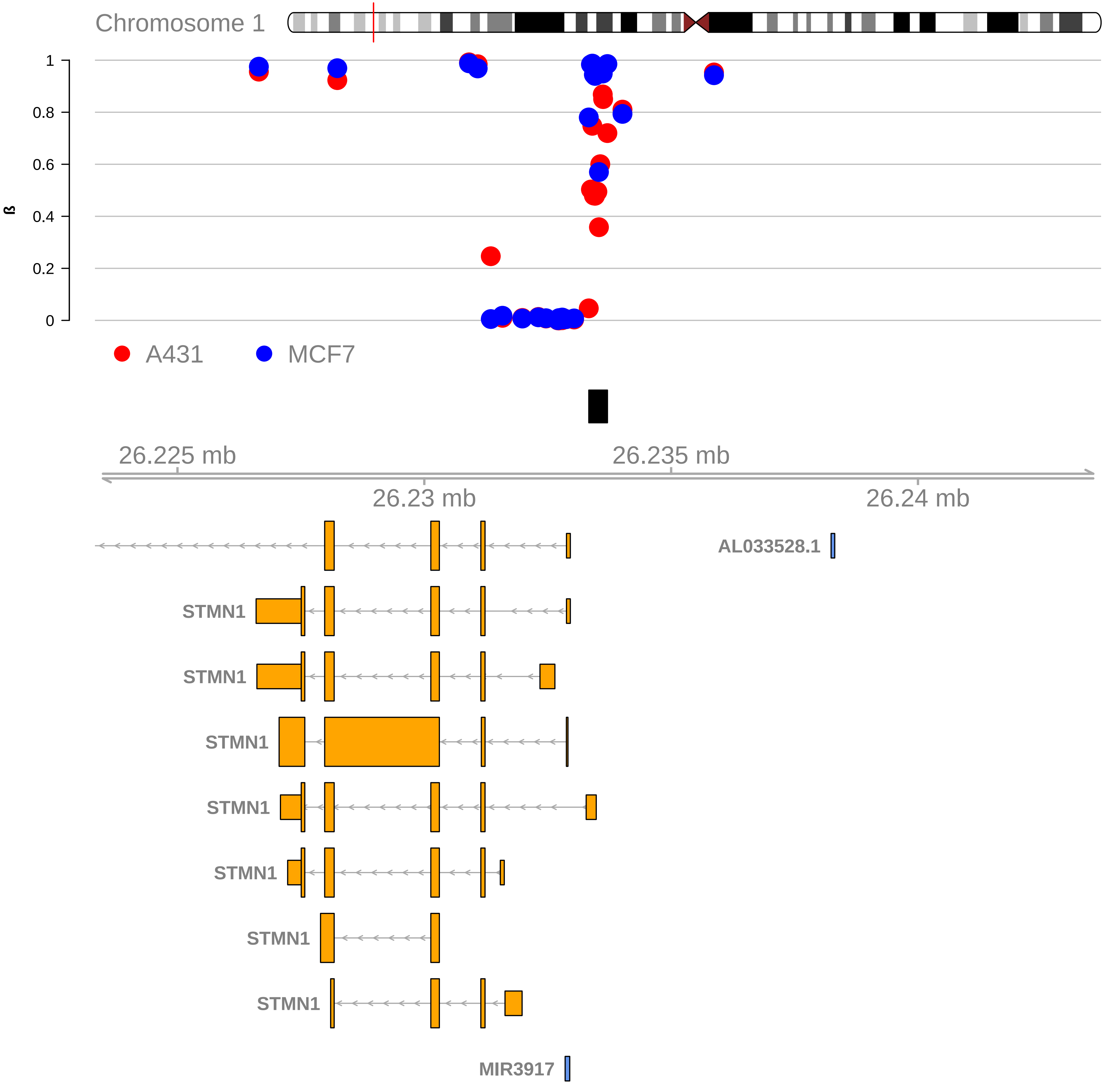

Chromosome 1
1
0.8
0.6
0.4
0.2
0
ß
A431
MCF7
26.225 mb
26.235 mb
26.23 mb
26.24 mb
AL033528.1
STMN1
STMN1
STMN1
STMN1
STMN1
STMN1
STMN1
MIR3917
